# The Innate Immune System Surveillance Biomarker p87 in African Americans and Caucasians with Small High-Grade Dysplastic Adenoma [SHiGDA] and Right-Sided *JAK3* Colon Mutations May Explain the Presence of Multiple Cancers Revealing an Important Minority of Patients with *JAK3* Mutations and Colorectal Neoplasia

**DOI:** 10.3390/gidisord6020034

**Published:** 2024-06-07

**Authors:** Martin Tobi, Xiaoqing Zhao, Rebecca Rodriquez, Yosef Y. Tobi, Tapan Ganguly, Donald Kuhn, Benita McVicker, Michael J. Lawson, John Lieb, Jaime L. Lopes

**Affiliations:** 1 Department of Research and Development, Detroit John D. Dingle VAMC, Detroit, MI 48201, USA; 2 Central Michigan University, Saginaw Campus, 1632 Stone St., Saginaw, MI 48602, USA; 3 Philadelphia VAMC, 3900 Woodland Avenue, Philadelphia, PA 19104, USA; 4 Department of Genetics, Perelman School of Medicine, Clinical Research Building 500, 415 Curie Blvd., University of Pennsylvania, Philadelphia, PA 19104, USA; 5 Department of Research and Development Service, Detroit VAMC, 4646 John R., Detroit, MI 48201, USA; 6 Research Service, VA Nebraska-Western Iowa Health Care System, The University of Nebraska Medical Center, Omaha, NE 68105, USA; 7 Department Gastroenterology, University of California, Davis Sacramento, 3160 Folsom Blvd., Suite 3500, Sacramento, CA 95816, USA; 8 Divisions of Gastroenterology, Hepatology and Nutrition, University of Florida at Gainesville, Gainesville VAMC, 1601 Southwest Archer Road, Gainesville, FL 32608, USA; 9 Cincinnati Children’s Hospital, Division of Genetics, Department of Pediatrics, University of Cincinnati, 3333 Burnet Ave., Cincinnati, OH 45229, USA

**Keywords:** adenoma, Adnab-9, African Americans, colorectal cancer, high-grade dysplasia, ion torrent, *JAK3*, Monoclonal antibody, p87

## Abstract

Colorectal cancer (CRC) outcomes in terms of incidence and mortality are significantly worse in African Americans than other Americans. While differences in primary preventions for neoplasia (diet, obesity remediation, aspirin prophylaxis) are being elucidated, genetic mutations affecting premalignant lesions and immune response mechanisms may possibly also explain the increased incidence and mortality, particularly from right-sided disease. Objective: Our team therefore examined colonic segments seeking to test the hypothesis that the immune response and somatic genetic profiles of the colonic anatomic segments may vary and thus account for variations in neoplasia risk among the various colonic segments revealing an antigenic relationship with precancerous lesions. The p87 antigenic field effect is recognized via Adnab-9 antibody immunohistochemistry to be significantly less in the right colon in African Americans, particularly in the cecum. Method: Since small high-grade dysplastic adenomas (SHiGDA) likely missed by CRC screening may progress to cancer, we used Ion Torrent^™^ sequencing of DNA extracted from four normal colonic segments (two left-sided and two right) of patients with SHiGDAs. We also contrasted unique mutational fields in one patient with a large HiGDA (*APC* with unique mutations) and one patient who prospectively developed a SHiGDA (*JAK3*). Result: The SHiGDA (small high-grade dysplastic polyp) patient was p87 negative for any extracted stool, saliva, or colonic effluent via ELISA (enzyme linked immunoadsorbant assay). Furthermore, mean values of expression in segments from the right colon were reduced with respect to the means obtained from the left segments in 233 patients evaluated for a p87 field effect. This has recently been shown to be the case in a large cohort of AA and Caucasian 2294 patients, possibly explaining the right-sided CRC disparity in African Americans and the subsequent increase in mortality. This field effect disparity is also true for two cancers contracted by the SHiGDa patient (lung and prostate). Conclusion: Thus, this pilot study suggests that the reduction in p87 in the right colon is possibly correlated with *JAK3* mutations. If confirmed, *JAK3* mutations, known to be associated with immune aberrations, may provide a mechanistic explanation for the lack of a p87 (protein 87 kilodaltons) field in some patients with HGD polyps who might benefit from possible intervention such as more intensive screening. Limited microbiome studies were also performed on two patients with familial cancer syndromes and these compared favorably with controls available from the literature.

## 1. Introduction

**Figure 1.**
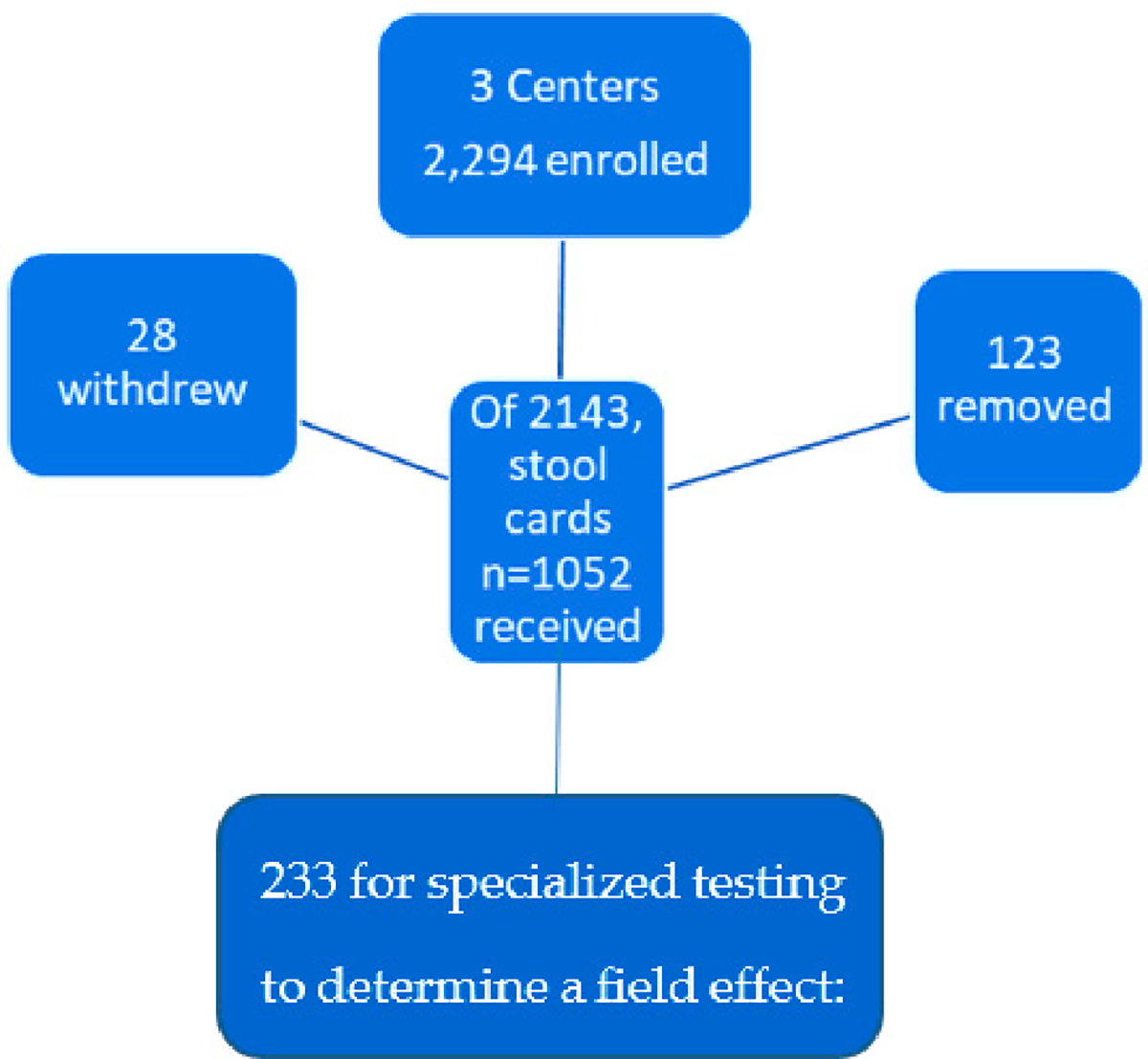
A graphic depiction of the patient pool from which the case report material was drawn.

Understanding definitive mechanisms of causation of severely dysplastic, small lesions would help direct CRC screening efforts in the African American patient population [1]. During a prospective, diagnostic screening study (see Figure 1 below) in populations at increased risk of CRC [2], we noted that some patients developed microscopic high-grade dysplasia within small adenomas (≤1 cm). This presents a clinical quandary in that the colonoscopist belatedly learns of the presence of high-grade dysplasia and the question of how to re-identify the site for complete removal arises since generally the location of smaller lesions is not tattooed.

To complicate matters, prospective colonoscopic randomized CRC screening trials have not shown a substantial reduction in CRC mortality [3]. Previous studies showed a reduction of >60% in right-sided CRC mortality compared to a >80% reduction in left-sided mortality [4–8] suggesting bimodal categories of tumor phenotypes. In African Americans (AA), this difference is magnified [9]. Whatever the explanation [10–19], this shortcoming may disrupt the screening effort in the population over age 45 and a new approach is needed. In future studies, next generation sequencing may be incorporated to reveal the genetic mutation we describe here.

## 2. Results

The veteran population is overwhelmingly male and a high-risk group for colorectal neoplasia. The demographic data are shown in Table 1.

Although the overall percentage of smokers was equivalent between AA and Caucasians, AA with a FE had a strong trend to smoke less (*p* < 0.08) and conversely more Caucasians with a FE were significantly more likely to smoke, suggesting that in this latter group, smoking may have invoked an increase in p87.

Initially, these patients who were enrolled in a prospective study and were selected based on past pathologic findings but after they were enrolled, the samples obtained were used mainly on the practical basis of availability and adequate high-quality DNA content. Most patients were at increased risk of CRC comprising 10 patients by virtue of past neoplasia, occult blood positive, or family history. Twelve phase 2 patients volunteering for additional FE mapping studies had HGD adenomas (five small and seven large) of whom eleven were also phase 2 NIPCON; phase 2 patients were included. The demographics are shown in Table 2. Approval for the studies was granted by the WSU School of Medicine IRB and Kaiser Permanente Medical Center where 14 patients (ten male and four female) also gave written informed consent.

One patient with an index colonoscopy which revealed a LHGDA and another with a SHiGDA found at a seven-year surveillance colonoscopy, were selected based on the availability of fresh frozen tissue extracts from the same four colonic regions (cecum, ascending, descending, and sigmoid colon) for extracting tissue, paraffin block biopsies, or serum DNA.

We found a specific *JAK3* mutation (c.394C > A) in the patient with the SHiGDA (see below). We therefore performed confirmatory PCR and found *JAK3* mutations in the gene product in tissue extracts from three patients (a female with Muir-Torre syndrome, a male AA with a 7 cm LHGDa, and a Caucasian male and female with APC in both serum and tissue extracts, collected respectively) that served as positive controls. The demographics of patients who served as a candidate pool of patients for positive controls for the *JAK3* mutation PCR, are summarized in Table 3 below.

The age differences were all statistically significant and clinically accounted for but there was only an insignificant trend between the ages of the HGD group (67.3 ± 10.3 large HGD and 61.2 ± 7.1; *p* = 0.28). Similarly, ethnic demographics were significantly different but these corresponded to the population from which they were drawn (cancer family syndrome vs. high risk {*p* < 0.04} and the latter vs. HGD {*p* < 0.02}). The presence of p87 antigen-expressing IHC fields in all but two (25%) of the SHiGDA patient’s colons were similar to the patient with the larger HGD polyp.

From the personal genome machine (PGM) sequencing, a total of about 3 million reads were obtained after initial filtration of polyclonal and low-quality reads from the dataset. The mean read length was 110 bases. Ninety-eight percent of the bases were aligned to human genome hg19 with a mean raw accuracy of 99% for 1Xreads. In Table 4 we show the coverage analysis of each barcoded sample. This includes on-target mapped reads, mean depth, and uniformity. These parameters were well within the range for similar samples run by the facility.

The coverage analysis is shown in Table 4 below.

The run (Table 4 summary) was therefore most satisfactory, showing high quality parameters and high feasibility for reproducibility as all native tissue DNA extractions performed were assayed for DNA quality. Table 5 lists the mutations found in the various samples ranked from the proximal right-sided (cecum and ascending) to distal [descending and sigmoid] colon.

There were a greater number of mutations in the SHiGDA patient. We confirmed the presence of the same *JAK3* exon 4 mutation demonstrated via the Ion Torrent run in the four colonic regions and examined and expanded these positive findings to include the transverse colon and rectum and found the mutation in a large polyp with HGD from an additional patient. When samples were run with commercially available exon 2 primers, no similarly specific mutations were found as expected and provided a negative control for the known mutations in exon 4.

While providing a testable hypothesis, we attempted to extrapolate these patients’ findings to a representative group for each HGD size group based on sample availability by using equivalent PCR primers to expand the patient sample size to test for the *JAK3* exon 4 mutation. The Ion Torrent data supported the existence of a genetic FE in these two patients.

In the large HGD polyp examined, the presence of mutated *KRAS* in the presumptive presence of wild type *TP53* was somewhat unusual but the technology may not necessarily detect all variants [20], such as large inserts and deletions. p53 alterations do not occur until the adenoma-to-carcinoma conversion. Also, it has been shown that KRAS mutations were preferentially found in larger adenomas, whereas p53 LOH occurs precisely at the adenoma-to-carcinoma transition [21]. Large insertions and deletions above 200–300 bps are not detected by next generation sequencetechnology which may explain this variable.

The high-grade dysplastic adenoma lesion found in Patient *A* harbored four likely driver mutations, including a novel somatic frameshift variant in *APC*, a novel missense SNP in *KIT*, and a novel missense SNP in *SMO*, in addition to a rare missense variant in *KRAS* (rs121913529) considered pathogenic for numerous cancers [22]. The *KIT* variant c.1672A > G found in this patient’s HGD lesion was also present in the normal mucosa of the descending colon in addition to a novel missense variant in *APC* indicating that these variants probably occurred at an earlier stage. In Patient B, all four samples from normal mucosa regions were found to be homozygous for the same novel *APC* frameshift variant in addition to a missense variant in *JAK3* (rs3212723). The ascending and sigmoid colon was also found to carry the same novel *KIT* variant (c.1672A > G) as in the normal mucosa descending colon of Patient A. In addition, the normal mucosa cecum also harbored the same novel missense *APC* variant as that found in Patient A. A summary of the mutations for novelty comparison is presented in Table 6.

**Figure 2.**
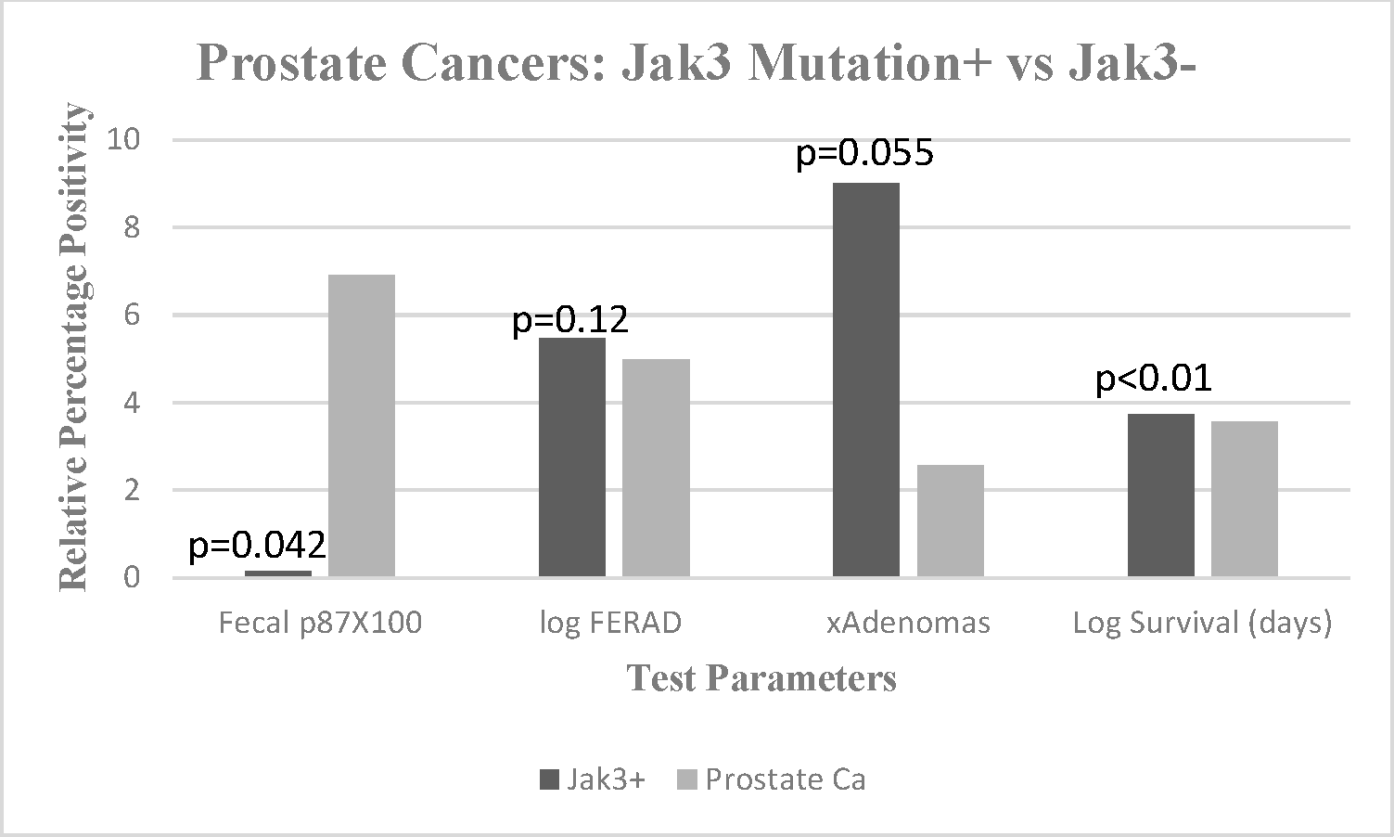
Effects of *JAK3* mutation on p87, FERAD ratio, neoplasia, and survival.

The markers revealed the presence of a specific genetic FE bearing an inverse relationship with a p87 field effect in the colonic mucosa of the SHiGDA patient. The nature of both genetic and antigenic FE appeared to be qualitatively and quantitatively different from the patient with the large lesion. We did, however, demonstrate that the means of the right and left sides for both ELISA and IHC were significantly lower on the right side in the two patients with the common *JAK3* mutation. This was consistent with the findings in the phase 2 NIPCON cohort as seen in Figure 2 and suggests a suppressive effect of the c.1672A > G *KIT* mutation. It is of interest that while these patients had different sized adenomas with HGD, neither expressed p87 in stool nor saliva (0 vs. 0.014 ± 0.013; *p* < 0.017 and 0 vs. 0.064 ± 0.034; *p* = 0.09), respectively. In order to summarize these and other findings, we contrasted three *JAK3*+ patients, all of whom had a history of prostate cancer with 50 prostate cancer patients from our database not known to be *JAK3*+. The data are shown on Figure 2.

The bar diagram shows significantly lower mean fecal p87 (black) compared to prostate *Jak3* negative controls (gray, at right) mean ± standard deviation OD (optical density) minus background (0.001 ± 0.000). Log FERAD ratio levels (p87:ferritin) tend to be higher in *JAK3*+ patients, mean ± standard deviation (292,133 ± 173,883 vs. 94,888 ± 162,290). Mean cumulative adenoma numbers also tended to be higher in *JAK3*+ patients mean ± standard deviation (9 ± 3.61 vs. 2.57 ± 5.42). There did seem to be a significant survival advantage with *JAK3*+ patients, mean ± standard deviation (5274 ± 370 vs. 3671 ± 1505).

We did not find significant differences in the control groups that may have predisposed to different colorectal neoplasia predisposition outcomes such as NSAID use; BMI; smoking; drinking; or chronic hepatitis.

There are no current *JAK3* mutations associated with microbiome studies aside from a solitary paper correlating skin microbiome with the Jak-Stat pathways [23]. In order to elucidate the presence of a *JAK3*-altered microbiome, we were able to define the microbiome in one APC patient with the seminal *JAK3* mutation and one patient with a variant of the Lynch Syndrome, the Muir-Torre Syndrome who did not have a *JAK3* mutation. We compared their microbiomes to 87 patients with Lynch syndrome and 10 APC patients from the literature. If the microbiome of one of our two patients significantly differed from the literature controls, we could hypothesize that the *JAK3* mutation would be implicated in at least the *JAK3*+ patient. If there was no perturbation observed and microbiomes between test and control patients were concordant, a *JAK3* mutation effect would be unlikely. Since multiple interventions for *JAK3* mutations are available, intervention would be theoretically possible to normalize the microbiome with modulation of the *JAK3* effects [24].

**Figure 3.**
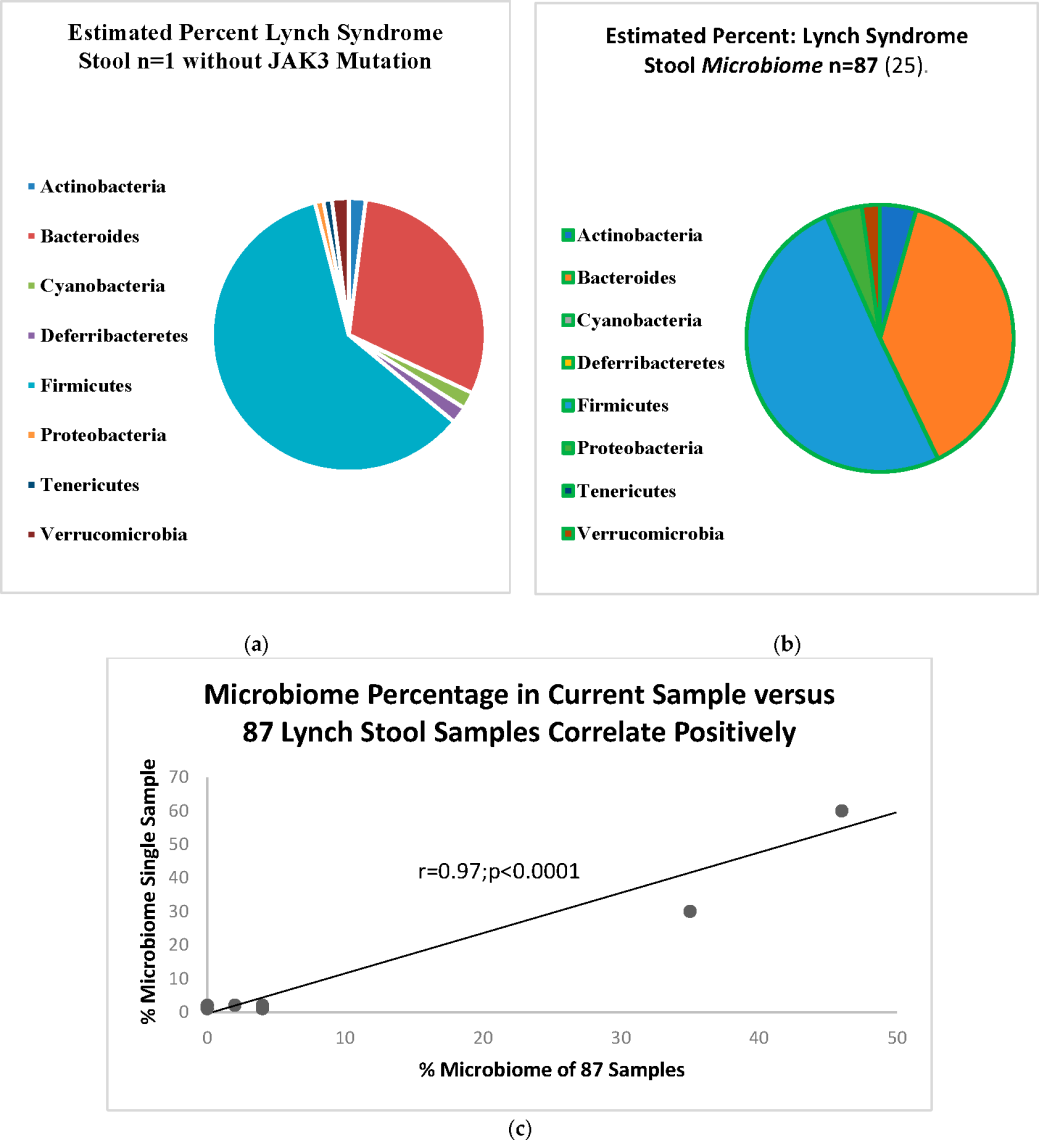
(**a**) Microbiome in Lynch Syndrome; (**b**) positive control microbiome; (**c**) regression analysis showing a significantly significant positive correlation of the Lynch microbiomes regardless of *JAK3* mutation.

Figure 3a shows the stylized pie-chart depiction of the microbiome in the Lynch syndrome patient as compared to the 87 Lynch syndrome patients in Figure 3b. The regression graphs show a significant positive correlation for the organisms tested as shown in Figure 3c.

**Figure 4.**
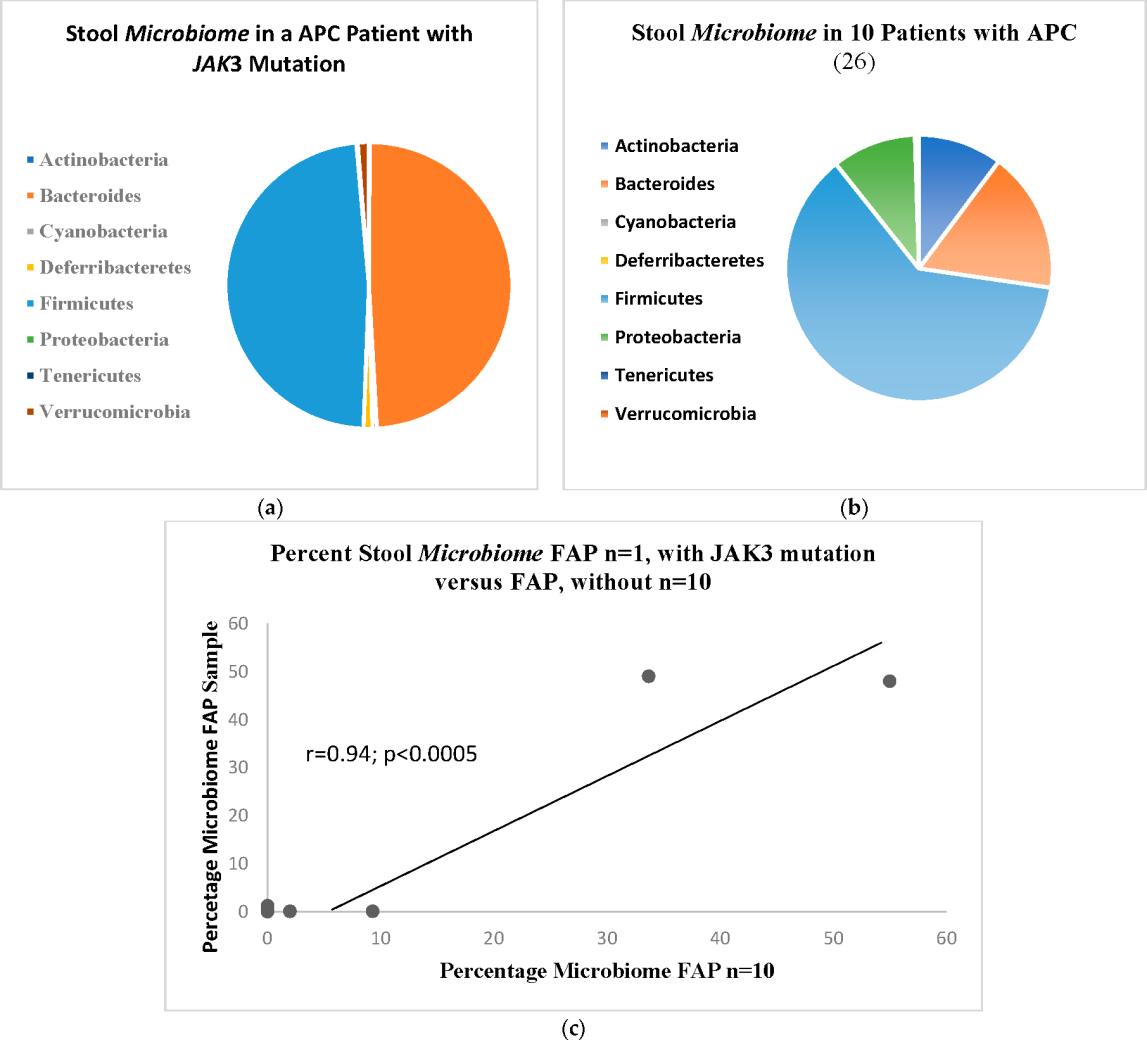
(**a**) APC patient with the JAK3 mutation; (**b**) distribution shown in 10 APC Controls. (**c**) Regression analysis in the correlation of APC patient with JAK3 compared to Positive APC controls.

**Figure 5.**
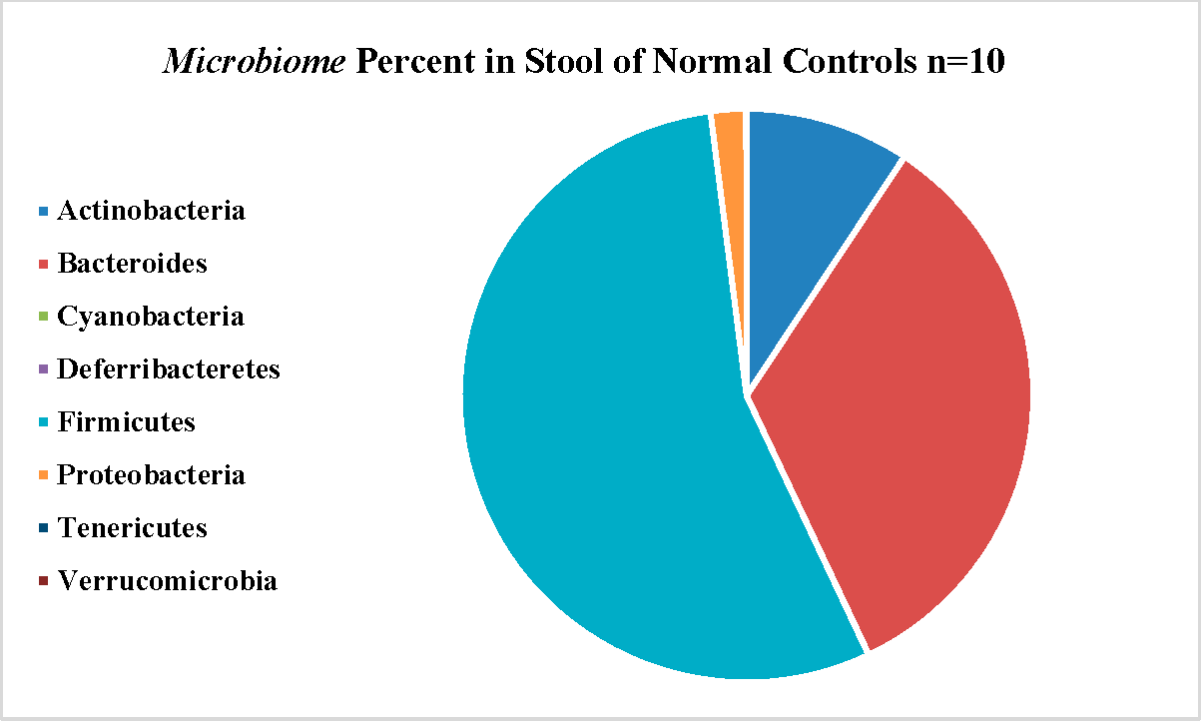
A normal distribution, also drawn from the literature is shown.

Figure 4a shows the microbiome bacillary distribution in the APC patient with the *JAK3* mutation compared to the distribution in 10 APC patients shown in Figure 4b with a similar correlation as above, shown in Figure 4c. The normal distribution, also drawn from the literature is shown in Figure 5.

Figure 4a shows the microbiome bacillary distribution compared to the distribution in ten APC patients shown in Figure 4b with a qualitative decreased change in the percentage of the bacteroides array with a complementary expansion of the firmicutes percentage.

## 3. Materials and Methods

Since many precursor adenomas may arise within a regional cancer field effect (FE), we have developed a monoclonal antibody (Adnab-9) that detects a p87 adenoma antigen labeling 76% of severely dysplastic colonic lesions [20]; it is also used to define a FE [25–27]. Adnab-9 ELISA testing in stool can predict up to 80% of adenomas [28]. This compares well to the latest results with a complex genetic-hemoglobin immunoassay for cancer detection (92%); for HGD polyps (69.2%); and 42% for other advanced adenomas [2]. Defining a molecular relationship to the Adnab-9 field in SHiGDA may enhance our understanding of direct interventions.

### 3.1. Patient Populations

A group of 2294 enrollees into the NIPCON study were evaluated for outcomes at colonoscopy after having provided a precolonoscopic stool sample for p87 testing. A subgroup of 233 consented to have additional studies (Phase 2) that allowed us to determine the presence of a p87 field effect [2]. In the entire group, 49 were found to have adenomas with HGD, 28 with large HGD polyps and 21 with SHiGDA. For relevant demographics please see Table 2. One representative from the large group and one from the small group underwent Ion Torrent mutation screening (see in Figure 1).

### 3.2. Ion Torrent^™^ PGM Sequencing

DNA was isolated using a QIaAmp DNA Kit (Qiagen, Valencia, CA, USA) from the normal tissue extracts obtained via endoscopic biopsy from the original study patients as described above. Samples of 10 ng DNA from fresh biopsies of cecum, ascending, descending colon, and rectum from each of the two patients, were analyzed via the Ion Torrent^™^ PGM sequencing at the University of Pennsylvania DNA Sequencing Facility. Briefly, barcoded AmpliSeq libraries used for the targeted sequencing of human genes were prepared from these eight samples obtained from colonoscopic biopsies and an additional sample extracted from the paraffin block of the large HGD polyp, to allow for the use of fixed tissues for future studies if successful, making a total of nine samples in all. The AmpliSeq Cancer Hotspot panel v.2 used in the study, following manufacturer’s instructions, includes 207 target regions for amplification ranging in size from 111 to 187 bp, covering 50 oncogenes and tumor suppressor genes previously implicated in cancer, and more than 2800 sequence variants described in the COSMIC database (Life Technologies, Carlsbad, CA, USA). The quality assessment of the barcoded libraries was performed via quantification with a picogreen assay on a Qubit fluorimeter (Invitrogen, Life Technologies, Waltham, MA, USA) followed by an Agilent BioAnalyzer run using a DNA high sensitivity chip (Agilent Technologies, Santa Clara, CA, USA). After equimolar pooling of nine libraries, the pooled libraries were diluted to 20 pM and were amplified on Ion Sphere Particles using the Ion OneTouch 200 system. Following enrichment to eliminate null beads, the sequencing was performed on a 318 chip in the Ion PGM sequencer (Life Technologies, Carlsbad, CA, USA, as above).

Sequencing data were analyzed with the Ion Torrent Suite v. 3.4 (Life Technologies). After barcode sorting, the reads were aligned to the human reference genome build 38 (hg19) using TMAP. Single nucleotide variants (SNV) and indels were detected using Variant Caller. The resulting variant caller files (vcf) were then annotated using Ion Reporter v. 2.2 (Life Technologies). The summary of the genetic mutations is shown above in Tables 5 and 6.

### 3.3. PCR Assays

To confirm and expand the scope of the findings, PCR was performed for *JAK3* mutations using primers which we had constructed commercially for this purpose (Integrated DNA Technologies, Coralville, IA, USA). For comparison, these PCR studies were also performed in two control FAP patients from whom DNA was available. DNA was extracted and purified from tissue extracts or serum using a spin protocol kit (Qiagen, Mansfield, MA, USA) and same-sourced QIaAmp Mini spin columns after proteinase K treatment. PCR was run with standard reagents on a Perkin Elmer Thermal PCR Cycler (Shelton, CT, USA) for 35 cycles. The table below shows the upstream and downstream primers used. PCR products were loaded onto 1.6% agarose gels at 5 ug/well and runs at 1 mA/cm^2^ for 1 h. The gels were stained by ethidium bromide 0.05% and the result band/s visualized under ultraviolet light using the EZ-run prestained Rec protein ladder (ThermoFisher BioReagents, Waltham, MA, USA). The predicted ~222 bp bands were then excised from the gel, eluted via precipitation with the easyDNA kit (Amersham Biosciences Corp., Piscataway, NJ, USA), and sequenced via the Genewiz sequencing service (South Plainfield NJ Suite 111, Shirley, NY, USA).

Left primer: JAK3exon4F 5’-AAGGTACAAGCTGGGCTCTG-3´ Integrated DNA Technologies

Right primer: JAK3exon4R 5´-TGAGGCCACCCAACTTCAAG-3´ Integrated DNA Technologies

### 3.4. Microbiome Determination

The methodology was quite standard and similar to those articles we cite [25,26]. Briefly purified DNA was obtained as described above which was sequenced allowing the taxa to be characterized via shotgun metagenomics methodology.

### 3.5. ELISA Testing and Immunohistochemistry

This was a standard sandwich antibody assay methodology and is described in detail in some of our publication cited here [27–29]. Currently no kit is available but components for the assay are commercially available. The fecal assay range generally ranges from 0 to 3 when read at 405 nm. Sensitivity was at 67% and 91% specificity for pancreatic cancers when prospectively determined and 80% and 87% retrospectively.

### 3.6. Data Analysis

Statistical analyses for parametric data were performed using the Student *t* tests and for ordinal data via Chi-square using a statistics software program graciously provided by VassarStats online website http://vassarstats.net/ (accessed on 13 December 2023).

## 4. Conclusions

While these data are informative, they were based on a relatively small number and we would like to have run *JAK3* PCR (polymerase chain reaction) on the control patients to have been assured of the group’s *JAK3* mutation status. We aim to apply these methods to a larger number of patients since this system is user-friendly, reliable, and most PCR-detected mutations are highly reproducible as is discussed below.

There are multiple studies of CRC in the African American population most recently reviewed by Carethers [23] showing that most studies show more proximal neoplasia and more advanced neoplasia on the right-side of the colon with greater lesion aggressiveness and poorer prognosis. Not all studies showed significant genetic changes in AA (African Americans) but more *KRAS* mutations and MSS CRC tumors were more prevalent [30]. These small polyps, starting as primordial tubular adenomas are the earliest macroscopic manifestations of neoplasia, and may progress to HGD (high grade dysplastic) lesions with significantly less transition through a villous morphology (Table 2). Of much interest is that the CIMP methylated serrated polyps in AA, the type most strongly held to be right-sided cancer precursors, are equivalent to the general population, suggesting that CIMP methylated polyps do not account for all the increased risk in the right side of the colon of African American patients. Tandem repeats associated with MSI (microsatellite instability) lesions are seen and they are targets for in/del mutations in the absence of pMMR (proficient mismatch repair) activity [29]. Our previous findings showing lower prevalence of right sided p87 FE are consistent with the generally observed location of CRN in AA suggesting that FE may be a protective feature [2]. BMI changes seen both in the literature [30] and our present study may also suggest changes in the microbiome also described in AA in the above review [2].

If confirmed, this may represent a paradigm shift [20] but this would not apply to the genetic field effect demonstrated in normal-appearing tissues of these two patients and larger numbers of patients would be required to resolve this issue. Also, the large lesion has an *APC* mutation in exon 16 resulting in a frameshift deletion. This was not seen in the corresponding regional normal mucosa but a distinct mutation in the same exon also resulting in a frameshift deletion was seen throughout the regional mucosa of the colon of the patient developing SHiGDA. Thus, a similar *APC* derangement was seen in both a large HGD lesion and within normal mucosa that ultimately produced SHiGDA. If confirmed, this might explain the tendency to more metachronous HGD lesions in SHiGDA patients and possibly affect the future development of cancer given the richer mutational milieu in SHiGDA-associated, colonic mucosa. Thus, the greater number of normal colon mutations in the patient later manifesting SHiGDA may belie an underlying genetic instability. The presence of late-stage markers in the SHiGDA patient was unanticipated as was the greater number of detected mutations in the colonic segments occurring at the putative onset of the dwell time-point [1] for the SHiGDA lesion, conferring a possible growth advantage.

Advanced colorectal tumors are marked by the accumulation of multiple driver mutations. It has been speculated that this is due to a multistep progression from adenoma to carcinoma and that identifying early drivers in the initiation of tumorigenesis could lead to earlier detection and more targeted therapies, improving clinical outcome such as a positive outcome with positive lesional p87 expression in CRC [31], similar to that of pancreatic cancer [32,33] However, the order in which these drivers occur is not well established. Through Ion Torrent sequencing on the normal mucosa of two patients with high grade dysplastic adenoma lesions, we observed somatic mutations in *APC*, *KIT* and *JAK3* in these prehigh-grade lesion loci.

Others [34] have focused on epidemiological evidence showing the decline in CRC incidence and mortality which began before the era of average-risk CRC screening. This decline has been less precipitous and lags significantly when compared to that of Caucasians. Other groups have successfully addressed patient and physician barriers and suggest screening in AA begin at age 45 [9]. Generally speaking, decreased p87 cancer tissue binding has been associated with poorer prognosis in CRC [31], IPMN (intraductal papillary mucinous neoplasm) [33], and pancreatic ductal adenocarcinoma [34]. Although not specifically noted in AA, the decreased antitumor cytotoxic immunity has been found to be deficient in AA with microsatellite-stable CRC [35,36] and TP53, P72R mutations. These have also shown to be a potential marker in AA destined to contract CRC [36] and might provide another tool to identify a subpopulation at risk in order to direct screening. Recently, miRNAs have been described and miR-182 implicated as a potential explanation of poorer prognosis as despite upregulation, its putative targets [FOX]1 and FOXO3A are reduced in cancers of AA [37,38]. We identified a nonsignificant trend to increased smoking in AA which may create an inflammatory environment implicated in many cancers and supported by genetic studies [39].

While BMI results were similar in both groups, there is an interesting previous report where AA with adenomas had higher BMI values as compared to those without [40], and we were interested to see if our study would confirm this. In 26.9% of the AA group no adenomas were found up to and including the time of the index colonoscopy. When the BMI of this group was compared to those with neoplasia the difference was of borderline significance [27.34 ± 5.18 vs. 29.77 ± 6.30; *p* = 0.051, respectively]. In contrast the Caucasians [17.54% never having had neoplasia], they did not show this difference [29.45 ± 5.54 vs. 29.15 ± 6.06; *p* = 0.69, respectively] and their BMI values approximated those of the majority AA neoplasia-positive group explaining the similarity of BMI values in the larger cohorts. AA tended to have more lung cancer [4.65% vs. 1.79%; *p* = 0.29]. As a likely predisposition to the latter, AA tended also to be more likely to smoke [42.7 vs. 30.77%; *p* = 0.12]. Since this was a cohort at high risk for CRC, the prevalence of index colorectal neoplasia was similar (21 in AA and 20.3% in Caucasians) and was consistent with the average adenoma number seen in Table 3. Interestingly the major smoking effect on the FE was mainly seen in the Caucasian group in this pilot study.

Our differential results with the p87 marker recognized via the Adnab-9 monoclonal antibody as related to the mutations detected may inform regarding SHiGDA etiology. *JAK3* mutations exclusive to SHiGDA suggest failure of immune surveillance by the gutassociated lymphoid tissue [GALT] to recognize the malignant potential of SHiGDA allowing unimpeded progress to infiltrative cancer. A recent paper on *JAK3* knockout mice and induced-colitis lends some support to this notion [41].

Paneth cells [2] are a component of the innate immune system that usually express p87, seen early in tumorigenesis. Since these cells are important in mucosal defense in eliminating potentially malignant cellular clones, we postulate that the absence of a field effect explains the escape of SHiGDA from immune surveillance. We postulate that p87 is expressed in response to the tumorigenic “pressure” as it seems to have the property of a physiologic acute-phase reactant (abstract) in reacting to temporary stress by a sharp but short-lived elevation. The data in Figure 3 suggest that *JAK3* mutations may partially explain the reduced fecal p87 and may explain the trend to a higher FERAD ratio. Since we believe that higher FERAD ratios are indicative of a more active InImS, this may have led to increased patient overall survival.

The *JAK3* variant is interesting as numerous malignancies have been characterized by excessive JAK activation. The JAK3 protein is involved in intracellular signal transduction via cytokine receptor and its activation in tumors is likely due to an increase in inflammatory cytokines. Clinical trials suggest that JAK3 small molecule inhibitors may be useful for growth suppression and antitumor immunity in solid tumor cancers [42]. The *JAK3* variant detected via the ion torrent sequencer seen in Patient B was considered conserved and occurred in a promoter and enhancer region and while rare in the European population (MAF = 0.0001), it had a MAF (minor allele frequency) of 0.14 in those of African descent (Table 6). Because colon cancer rates are notably higher in people of African descent [24,30], there is great interest in uncovering genetic predisposition loci in present in this population. Given the relative paucity of specific genetic changes described thus far in the African American population, *JAK3* mutations as we described as already disproportionately prevalent in this population may explain poorer clinical outcomes from the “host” side of the survival factor equation. Therefore, functional analysis of this missense variant in *JAK3* kinase activity would be worthwhile and most impressive pending additional data.

The results of the sequencing analysis in the patient with the large HGD adenoma, indicated that *APC* (Adenomatous Polyposis Coli), *KIT,* and possibly *JAK3* were the most likely early drivers of tumorigenesis in these patients. The observation that the *KRAS* mutation was absent in the normal mucosa was not controversial and supports current belief that *KRAS*-activating mutations occur later in the adenoma-carcinoma progression model, after *APC* driver mutations [43]. APC regulates WNT (wingless-related integration site) pathway signaling and has been implicated in FAP, an autosomal dominant genetic condition that typically leads to colorectal cancer if left untreated. KIT (proto-oncogene tyrosine-protein kinase) is a transmembrane receptor for mast cell growth factor and found to be constitutively active in 75% of gastrointestinal stromal tumors (GIST). The variant uncovered in our analysis which both patients share, occurs in exon 11. This locus is a hot spot for pathogenic variants for GIST as it contains the KIT juxtamembrane domain [44–47] and subvariants of CRC [47]. Recently, it was also shown that low-dose JAK3 inhibition may become an effectual immunotherapy option [42]. Dual therapies have been shown to effectively target CRC with *JAK3* and KRAS [43].

We would not have anticipated a *JAK3* mutation in this clinical scenario but we did find it in the serum of a surgically-resected FAP patient and another female patient with a somatic *JAK3* mutation, serving as positive controls for our PCR assay. While the polyp number was drastically reduced in patients’ remnant colon, they remain at risk of CRC and endoscopic surveillance is mandatory. This FAP patient’s clinical course was somewhat unusual and given his JAK3 mutation it is tantalizing to suggest that this may have played a role. We have shown that a small but definable proportion of patients with adenomas with HGD do carry this mutation. Since it was also found in the serum of the FAP patient in question, we suggest that it may also be a germline mutation but this remains to be conclusively proven. The fact that it is also found in FAP patients in the same proportion suggests that it may also play a role in the progression of tumorigenesis in these patients. Interestingly, a study with Min mice with a FAP phenotype derived benefit from anti-JAK3 therapy [41] suggesting that FAP patients with the *JAK3* mutation may also benefit from this treatment option. While ongoing studies to prove this hypothesis are envisaged, we do not desire to overinterpret this chance encounter.

### Pitfalls and Promise

In summary, while this study is small and limited in patient material, it does provide an in-depth view of the panoply of genetic mutations beneath the surface of what appears to be “normal” mucosa and this has the potential to change the paradigm of thought in future research. We have shown that *JAK3* mutations are not just confirmed in a small subset of AA but ostensibly affect other individuals and even those with additional *APC* mutations, although the exact nature of the perturbation caused by this mutation remains to be elucidated. What is clear is that we have the technology to block *JAK3* mutations and their co-conspirator genes. This makes it imperative that we act quickly to screen for these mutations via colonoscopy sampling of both lesions and “normal” mucosa, revealing the underlying mutational FE, and inactivating or blocking these specific mutations. This could prevent not just only CRC proliferation, but also enable a reduction in secondary cancers. This would be a true example of personalized medicine.

## Figures and Tables

**Table 1.** Demographics of Phase 2 NIPCON (noninvasive prediction of colonic neoplasia). Patients from which patient and controls were drawn.

Group	African American n = 119	Caucasian n = 114	*p*-Values

Age mean ± sd [yrs]	60.73 ± 10.61	59.98 ± 12.87	0.62
% Male	88.1	84.5	0.46
BMI	29.25 ± 6.08	29.21 ± 5.98	0.96
% GI symptoms	60.5	46.6	OR1.76 [CI 1.06–2.9] < 0.04
Average Adenoma #	1.91 ± 3.29	1.93 ± 2.80	0.97
%Family History CRC	16.8	10.5	0.19
% Smokers (%FE + FE−)	51.1 (35.3 vs. 47.5)	47.1 (71.4 vs. 30)	0.5 (AA0.075:Cau < 0.00005) *

GI-gastrointestinal

# -number; CRC-colorectal cancer; FE-field effect

* OR5.83 [CI 2.45–13.89].

**Table 2.** Demographics and data of patients with high grade dysplastic polyps from which Detroit patients were drawn.

HGD	N	Age yr	%AA	%Left	%TA	Size mm	%Smoke	#Syn	FOBT + %

Large	28	66 ± 8.9	70	21	44	20.2 ± 8.1	67	1.4 ± 1.3	67
Small	21	63 ± 10.1	29	33	79	7.4 ± 2.7	30	2.5 ± 2.1	20
*p* value	N/A	0.33	<0.01	0.54	<0.04	N/A	0.11	0.43	<0.05

Yr-Year; TA-tubular adenoma; Syn-synchronous neoplasia; derivation of p87 expression in two candidate patients, N/A-not applicable.

**Table 3.** Demographics and JAK3 detected proportions in the PCR groups.

Group	Family Cancer Syndrome	High Risk and Others	High Grade Dysplasia

Number	3	6	10
Age mean ± sd (yrs)	43.7 ± 7.0 *	58.2 ± 8.7	55.7 ± 11.4
% Male	33.3	85.7	100
% African American	0	16.7	66.7
% JAK3 c.394C > A	14.3	0	16.7

* Family cancer syndrome vs. HGD ages were significant at *p* < 0.043; other differences were not statistically significant. Other JAK3 mutations found were: c.346 A > C; c.431 G > A; c.431C > A; and c.359G > A. The differences between the FERAD ratios (ferritin/fecal p87) as reported in [2] showed a moderate trend between *JAK3*+ mutation patients and those patients in whom no *JAK3* mutations were detected as can be seen in Section 3.

**Table 4.** Coverage analysis and the number of variants for each barcoded sample.

IonXpress Code	Mapped Reads	On Target	Mean Depth	Uniformity	Variants	Hotspot Variants

NipCon_001 Large	98,792	95.95%	423.4	99.88%	20	7
NipCon_002	476,704	87.74%	1932	100.00%	16	5
NipCon_003	515,585	87.35%	2079	100.00%	16	5
NipCon_004	283,449	89.56%	1169	99.57%	18	6
NipCon_005	619,529	92.56%	2651	99.53%	14	4
NipCon_006	271,188	91.03%	1129	100.00%	19	6
NipCon_007	260,021	91.85%	1089	100.00%	18	6
NipCon_008	194,255	79.3%	712.8	100.00%	17	5
NipCon_009	212,251	90.40%	995.2	100.00%	17	5

**Table 5.** Summary of the distinct genetic mutations (nonsynonymous and indels) revealed via Ion Torrent^™^ assay.

IonXpressCode	Size HGD	Location	Distinct Mutation	#Mut	%ACS *	ACS Phase

NipCon_001 *	Large lesion	Descending	*APC, KRAS, KIT, SMO*	4	50	Early, mid
NipCon_002	Large	Cecum	*TP53, KIT*	2	50	Late
NipCon_003	Large	Ascending	*APC, TP53*	2	100	Early, late
NipCon_004	Large	Descending	*TP53*	1	100	Late
NipCon_005	Large	Sigmoid	*TP53*	1	100	Late
NipCon_006	Small	Cecum	*APC, TP53, PIK3CA, JAK3*	4	50	Early, late
NipCon_007	Small	Ascending	*APC, TP53, PIK3CA, JAK3*	4	50	Early, late
NipCon_008	Small	Descending	*APC, TP53, PIK3CA, JAK3, KIT*	5	40	Early, late
NipCon_009	Small	Sigmoid	*APC, KIT, PIKCA, JAK3*	4	25	Early

* Paraffin-embedded block DNA-rest fresh tissue; ACS-adenoma carcinoma sequence; % are proportion of ACS-associated genes (early, mid, or late). Other than the large lesion, all other extracts were from normal-appearing mucosa.

**Table 6.** Summary of mutations seen in large HGD lesion and in normal-appearing mucosa.

Sample	Gene	Variant	Result	AA	Zygosity	rsID	MAF AF	MAF EUR

Patient A								
HGDpol	*APC*	c.3950_3956delAAGATCC	frameshift	nonsense	HET	novel	N/A	N/A
HGDpol	*KIT*	c.1672A > G	missense	pLys558Glu	HET	novel	N/A	N/A
HGDpol	*KRAS*	c.35G > T	missense	p.Gly12Val	HET	rs121913529	0.0001	1.87 × 10^−5^
HGDpol	*SMO*	c.1886G > A	missense	p.Arg629Lys	HET	novel	N/A	N/A
Cecum	*APC*	c.4744G > A	missense	p.Ala1582Thr	HET	novel	N/A	N/A
Descend	*KIT*	c.4744G > A	missense	p.Lys558Glu	HET	novel	N/A	N/A
Patient B								
Cecum	*APC*	c.4744G > A	missense	p.Ala1582Thr	HET	novel	N/A	N/A
Cecum	*APC*	c.4479_4480delGG	frameshift	nonsense	HOM	novel	N/A	N/A
Cecum	*JAK3*	c.394C > A	missense	p.Pro132Thr	HET	rs3212723	0.14	0.0001
Ascend	*APC*	c.4479_4480delGG	frameshift	nonsense	HOM	novel	N/A	N/A
Ascend	*JAK3*	c.394C > A	missense	p.Pro132Thr	HET	rs3212723	0.14	0.0001
Ascend	*KIT*	c.1672A > G	missense	p.Lys558Glu	HET	novel	N/A	N/A
Descend	*APC*	c.4479_4480delGG	frameshift	nonsense	HOM	novel	N/A	N/A
Descend	*JAK3*	c.394C > A	missense	p.Pro132Thr	HET	rs3212723	0.14	0.0001
Sigmoid	*APC*	c.4479_4480delGG	frameshift	nonsense	HOM	novel	N/A	N/A
Sigmoid	*JAK3*	c.394C > A	missense	p.Pro132Thr	HET	rs3212723	0.14	0.0001
Sigmoid	*KIT*	c.1672A > G	missense	p.Lys558Glu	HET	novel	N/A	N/A

Footnote to table: Ascend = ascending colon; Descend = Descending colon; HGDpol = large high-grade dysplastic polyp; HET = heterogenous; HOM = homogenous; MAF = minor allele frequency for African Americans MAF EUR minor allele frequency for non-Finnish Europeans (information obtained through the ExAC@C database-Oracle Exadata Cloud at Customer website:https://search.yahoo.com/search?fr2=p:ds,v:omn,m:sa,brws:chrome,pos:5&fr=mcafee&type=E210US739G0&p=exac+database+system(accessed on 25 February 2016, Thursday)).

## Data Availability

The data presented in this study are available on request from the corresponding author due to the unpublic data.
